# The advances of adjuvants in mRNA vaccines

**DOI:** 10.1038/s41541-023-00760-5

**Published:** 2023-10-26

**Authors:** Chunyuan Xie, Ruhui Yao, Xiaojun Xia

**Affiliations:** State Key Laboratory of Oncology in South China, Collaborative Innovation Center for Cancer Medicine, Guangdong Provincial Clinical Research Center for Cancer, Sun Yat-sen University Cancer Center, Guangzhou, 510060 China

**Keywords:** Adjuvants, RNA vaccines

## Abstract

The remarkable success of messenger RNA (mRNA) vaccines against severe acute respiratory syndrome coronavirus 2 (SARS-CoV-2) has propelled the rapid development of this vaccination technology in recent years. Over the last three decades, numerous studies have shown the considerable potential of mRNA vaccines that elicit protective immune responses against pathogens or cancers in preclinical studies or clinical trials. These effective mRNA vaccines usually contain specific adjuvants to obtain the desired immune effect. Vaccine adjuvants traditionally are immunopotentiators that bind to pattern recognition receptors (PRRs) of innate immune cells to increase the magnitude or achieve qualitative alteration of immune responses, finally enhancing the efficacy of vaccines. Generally, adjuvants are necessary parts of competent vaccines. According to the existing literature, adjuvants in mRNA vaccines can be broadly classified into three categories: 1) RNA with self-adjuvant characteristics, 2) components of the delivery system, and 3) exogenous immunostimulants. This review summarizes the three types of adjuvants used in mRNA vaccines and provides a comprehensive understanding of molecular mechanisms by which adjuvants exert their functions in mRNA vaccines.

## Introduction

The mRNA vaccines have emerged as a promising vaccine format compared to conventional vaccines because of their advantages: high potency, rapid development, safe administration, and low-cost manufacture^1^. Since the 1990s, researchers have made successive attempts to use mRNA to produce target proteins and develop mRNA vaccines against infectious diseases in preclinical models^2,3^. Nowadays, mRNA vaccines have shown great potential in many preclinical and clinical studies against multiple diseases, such as autoimmune encephalomyelitis, tumors, infectious diseases including Zika virus disease, influenza A, coronavirus disease 2019 (COVID-2019), rabies, and so on^4–7^. It’s well known that mRNA vaccines have protected millions of people from the threat of SARS-Cov-2 because of their superior efficacy. This unprecedented success has proved that mRNA vaccines are safe, reliable, and potent weapons against infectious diseases. Interestingly, two recent clinical studies have shown that mRNA vaccines based on individualized neoantigens can potentiate the therapeutic efficacy of immunotherapy drugs like pembrolizumab and atezolizumab and delay the recurrence of melanoma and pancreatic cancer via inducing neoantigen-specific CD8^+^ cell responses^8,9^. These studies further highlight the promising future of mRNA vaccines in treating malignant diseases.

The mRNA vaccine usually comprises three parts: the synthetic RNA molecules that direct the production of antigens, the material for mRNA delivery, and the adjuvant component. Adjuvants generally are additional immunostimulants besides nonliving antigens in vaccines, which activate innate immunity and provide the “help” needed to enhance the magnitude and quality of the adaptive responses to provide maximum protection against specific pathogens^10^. Different adjuvants tend to induce various immunological reactions that can tailor the outcome of vaccines. For example, commercialized adjuvant AS03 prefers to stimulate a mixed T helper-1 (Th1) and Th2 cell response, while adjuvant AS37 stimulates a Th1-biased response and adjuvant alum induces a Th2-biased response^11^. It is important to note that mRNA vaccines should produce customized immune responses in order to treat different diseases. For instance, strong T follicular helper (T_FH_) cell and germinal center (GC) B cell responses are needed to induce high titers of neutralizing antibodies against the infectious diseases caused by viruses^12^. On the other hand, treating other diseases such as tumors and malaria needs potent antigen-specific T cell responses to produce cytotoxic cytokines like interferon-γ (IFNγ) to kill target cells^13,14^. One feasible way to modulate the immune responses elicited by mRNA vaccines is to use different adjuvants to tailor the immune responses and thus meet the therapeutic requirements of treating different diseases. For example, an ionizable cationic lipid DLinDMA can act as a potent adjuvant for mRNA vaccines to induce robust T_FH_ cell and GC B cell responses to generate neutralizing antibodies, while Toll-like receptor 7/8 (TLR7/8) agonist R848 can elicit an effective anti-tumor immunity by significantly enhancing the antigen-specific CD8^+^ T cells responses^15,16^. Agonists for innate immune pathways beyond TLRs have been under test for their adjuvant activities in therapeutic vaccines for diseases with the complex immune microenvironment, such as tumors^17,18^. Agonists for the stimulator of interferon genes (STING) pathway, like CF501 and ADU-S100, exhibited potent adjuvant effect for mRNA vaccines in preclinical and clinical studies^19,20^. Ligands for the C-type lectin receptors pathway or nucleotide-binding oligomerization domain (NOD)-like receptors (NLRs) pathway also show robust adjuvant activity and deserve further investigation^21,22^. To summarize, adjuvants can influence the immune response types of mRNA vaccines and determine whether to improve the level of neutralizing antibodies or enhance antigen-specific cytotoxicity. Thus, it is vital to deepen our understanding of adjuvants in mRNA vaccines, which could provide valuable insights for designing next-generation mRNA vaccines.

Despite the rapid development of mRNA vaccine technology, there still is a lack of clear characterization of the adjuvants used in mRNA vaccines. Here, we overview the adjuvants used in mRNA vaccines in recent years by summarizing their common features and the relevant molecular mechanisms, expecting to improve the understanding of mRNA vaccine adjuvants for better vaccine design and preparation.

### Adjuvants based on the intrinsic characteristics of mRNA

**Fig. 1:**
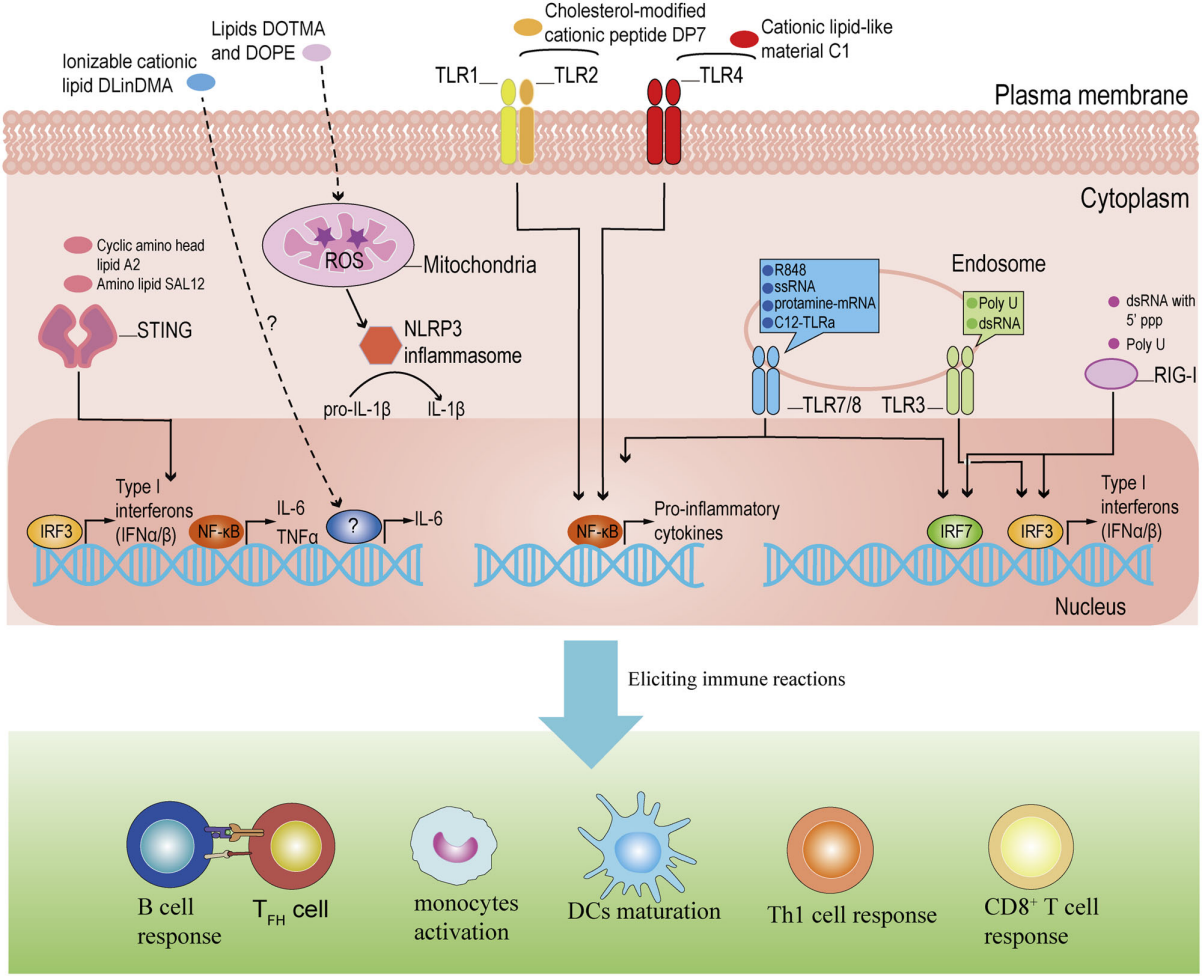
Adjuvants activate the signaling in mRNA vaccines. Adjuvants often act as ligands for PRRs in innate immunity to activate corresponding immune signaling pathways. These activated signaling pathways can promote the secretion of cytokines like IL-12, IL-1β, and IFNα/β and ultimately improve the immune effect of vaccines. IRFs, interferon regulatory factors; NF-κB, nuclear factor kappa light chain enhancer of activated B cells; ROS, reactive oxygen species; NLRP3, NOD-, LRR- and pyrin domain-containing protein 3; 5’-ppp, 5’ triphosphate.

Adjuvants often are a kind of immunostimulatory agent that elicit immune responses by binding with PRRs of innate immunity to enhance the efficacy of vaccines. It is well known that foreign RNA molecules have the potential to induce immune responses in mammalian cells. Exogenous nucleoside-unmodified mRNA used to express antigens in the mRNA vaccines has intrinsic adjuvant activity by triggering innate immune signaling. In particular, double-stranded RNA (dsRNA) can activate TLR3, single-stranded RNA can activate murine TLR7, and RNA oligonucleotides with phosphorothioate internucleotide linkages are ligands for human TLR8^23^. Poly uracil (U) and short dsRNA with 5’ triphosphate at the blunt end could act as adjuvants for mRNA vaccines by enhancing the immune responses through TLR3 and retinoic acid-inducible gene (RIG)-I signal pathways without hampering the antigen expression^24,25^. The activation of TLRs and RIG-I signaling could induce the production of proinflammatory cytokines such as tumor-associated factor-α (TNF-α), interleukin 6 (IL-6), IL-12, IL-1β and IFNα/β (Fig. 1), which on one side strengthen the desired protective immunity of mRNA vaccines, but on the other side may cause excessive inflammation^18^. The seminal work by Kariko et al. found that RNA molecules without nucleoside modifications activated TLR or RIG-I signaling to evoke antiviral-like immune responses, which may impair RNA translation and lead to RNA degradation^26^. Such immune activation can be avoided by nucleoside-modified mRNA, such as pseudouridine, which has been widely used in mRNA vaccines^23,27^. A recent study showed that the modified mRNA in the BNT162b2 mRNA vaccine from Pfizer-BioNTech may be recognized by melanoma differentiation-associated protein 5 (MDA-5) to trigger the production of IFNα, finally contributing to the magnitude of antigen-specific T cell and antibody responses^28^. It is intriguing that the host interferon responses in most scenario suppress mRNA translation and induce RNA degradation, but in some other cases promote antigen-specific responses induced by mRNA vaccines. Further work is warranted to delineate the underlying mechanism. Overall, RNA molecules in the mRNA vaccines can have adjuvant activity via intrinsic immune-stimulating characteristics, which should be considered when designing mRNA vaccines.

Aside from the intrinsic self-adjuvant characteristic of ribonucleotide sequences, mRNA can be used for producing functional immune proteins such as cytokines and antibodies to promote antigen presentation and improve vaccine efficacy. Antigen-presenting cells (APCs) are critical for vaccinology, but how to efficiently deliver antigens to APCs has always been challenging. Fc receptors are widely expressed on APCs to recognize and bind with the Fc portion of antibodies^29^. Therefore, making antigens fused with the Fc portion is a helpful method to deliver the antigens to APCs. Recently, there has been a report that an mRNA vaccine encoding the human Fc-conjugated receptor binding domain of SARS-CoV-2 (RBD-hFc) can efficiently prevent SARS-CoV-2 infection in the K18 promoter-driven human angiotensin-converting enzyme 2 (K18-ACE2) transgenic mouse model^30^. In another study, a constitutively active mutant of STING encoded by mRNA was used as an adjuvant to amplify antigen-specific CD8^+^ T-cell responses induced by mRNA vaccine to inhibit TC-1 tumor growth^31^. In addition, restoring the immunological fitness of the tumor microenvironment by mRNA encoding immune stimulatory molecules and immune checkpoint blockade antibodies is also under test in mRNA cancer vaccines^17^. For example, TriMix is an mRNA cocktail composed of three mRNA molecules encoding CD40L, CD70, and a constitutively active form of TLR4, which is currently under clinical trials as an mRNA therapy in dendritic cells (DCs) vaccine to prevent melanoma progression^32,33^. The mRNA-encoding bispecific T-cell engager (BiTE) is another promising therapy for eliminating advanced tumors, which encodes a fusion protein with an antibody targeting CD3 on one fragment antigen-binding arm and a tumor antigen on the other arm to induce sustained endogenous synthesis of the bispecific T cell-engaging antibodies^34^. Therefore, using mRNA to produce antigens together with specific functional immune proteins as adjuvants, such like STING protein and anti-CD3 antibody, is a feasible way to boost the efficacy of mRNA vaccines. For mRNA vaccines in cancer immunotherapy, directing mRNA to produce immune modulators like antibodies against programmed cell death 1 (PD-1) may also help amplify the mRNA vaccine efficacy, which deserves further study.

### Adjuvants based on the components of the delivery system

Due to the physiochemical features like biological macromolecule and negative charge, naked mRNA cannot efficiently enter the cells. Delivery tools are required to facilitate the transfer of mRNA into the cytoplasm for protein translation. Although there are a number of methods to promote the cytoplasmic entry of mRNA, lipid nanoparticle (LNP) has recently emerged as the optimal delivery material in this field^35^. LNP is often comprised of cationic lipids that form the complex with negatively charged mRNA and structural “helper” lipids such as cholesterol, phospholipids, or polyethylene glycol (PEG)-conjugated lipid^17^. Some lipid components in the LNP system have immunostimulatory characteristics that can act as adjuvants for mRNA vaccines (Table 1). Specifically, a lipid-coated calcium phosphate nanoparticle can act as an artificial nanosized Ca^2+^ reservoir to promote DC maturation by upregulating co-stimulatory molecules like CD80 and CD86 via providing extra Ca^2+^ in the cytoplasm^36^. In addition, the cationic lipid components may be crucial for the adjuvant activities of LNP. The LNP based on ionizable cationic lipid DLinDMA is immunostimulatory and can act as an adjuvant for nucleoside-modified mRNA vaccines to elicit potent T_FH_ cell responses and GC B cell responses with abundant neutralizing antibodies^15,37,38^. Intriguingly, although the adjuvant activity of DLinDMA relies on IL-6 production instead of MyD88 or MAVS-dependent signal pathways, there is still a poor understanding of the specific molecular mechanism by which it triggers the production of IL-6^15^. In addition, cationic lipid-like material C1 can deliver mRNA into the intracellular space, promote the release of inflammatory cytokines such as IL-1β, IL-6, and IL-12P70, and upregulate the expression of co-stimulatory molecules through the TLR4 signaling pathway^39^. Lipidoid C12-TLRa (composed of Epoxide C12 and amine-containing TLR7/8 agonist) can promote mRNA vaccine delivery and TLR response, together inducing high levels of neutralizing antibodies^40^. Another ionizable lipid-like material used for mRNA vaccine, A2-Iso5–2DC18 (A2), is composed of an unsaturated lipid tail, a dihydroimidazole linker, and a cyclic amine head group, which can activate STING signaling (Fig. 1) and the release of cytokines like C-X-C motif chemokine 10 (CXCL10), type I IFNs to enhance immune responses^41^. A non-nucleotide STING agonist-derived amino lipid, SAL12, can also formulate into LNP for mRNA delivery and induce the production of IFNβ to elicit potent neutralizing antibodies against SARS-Cov-2^42^. Dimethyldioctadecylammonium (DDA) is a quaternary ammonium lipid that can conjugate with mRNA and elicit innate immunity and Th1 cell responses, thereby serving as a potent immune adjuvant for mRNA vaccines against rabies virus^43^. However, the inherent immune stimulatory features of the lipid materials used for mRNA vaccines are not always beneficial for the vaccine. A recent study reported that the lipid components (DOTMA and DOPE) instead of unmodified mRNA in mRNA vaccines would promote the generation of mitochondrial ROS in monocytes to activate NLRP3 inflammasome and release IL-1β, triggering the inflammatory side effect of mRNA vaccine in human beings^44^. These studies emphasize the importance of the independent immune-stimulatory effect of lipid components. Hence, screening suitable and feasible lipid components with proper immune activation function is valuable for the rational design and development of future mRNA vaccines.

**Table 1:** The lipids and lipid-like materials with self-adjuvant activities in mRNA vaccines.

Name	Component	Proposed functions	References
LCP	An anionic lipid (DOPA) and calcium phosphate	An artificial nanosized Ca2+ reservoir to promote DCs maturation	Wang et al. 2018
DLinDMA	Ionizable cationic lipid	Depending on IL-6 signaling, this lipid can help the T_FH_ cell and germinal center B cell responses to generate abundant neutralizing antibodies.	Morrissey et al. 2005; Pardi et al. 2018; Alameh et al. 2021
C1	Cationic lipid-like material	Acting as the ligand for TLR4, C1 could promote the activation of DCs and cytokines like IL-6 and IL-12P70 to enhance immune responses	Zhang et al. 2021
A2	Ionizable cationic lipid-like material	Inducing the activation of STING signaling, this lipid could promote the release of CXCL10 and IFNs	Miao et al. 2019
DDA	A quaternary ammonium lipid	Enhancing the innate immunity and Th1 cell responses	Lou et al., 2020
DOTMA and DOPE	Cationic lipid and helper lipid	Elevating the mitochondrial ROS to activate NLRP3 inflammasome and release IL-1β to trigger innate immunity activation	Tahtinen et al. 2022
C12-TLRa	Lipidoid	Enhancing mRNA delivery and increasing the innate immunity responses with TLR7/8-agonistic activity	Han et al. 2023
SAL12	Ionizable amino lipid	Activating the STING pathway to produce IFNβ when delivering mRNA	Zhang et al. 2023

*LCP* Lipid-coated calcium phosphate, *DOPA* dioleoylphosphatydic acid, *DLinDMA* 1,2-dilinoleyloxy-n,n-dimethyl-3-aminopropane, *DDA* Dimethyldioctadecylammonium, *DOTMA* trimethyl[2,3-(dioleyloxy)propyl]ammonium Chloride, *DOPE* 1,2-Dioleoyl-sn-glycero-3-phosphoethanolamine.

In addition, the physical properties may influence the immunological activity of adjuvants. Recently, a study reported that the particulate form instead of the soluble form of mannans can act as adjuvants in vaccine formulations directed against respiratory viruses^45^. Moreover, a pathogen-mimicking hollow nanoparticle displaying mannan can activate the Dectin-2 and TLR4 receptors in DCs, and induce the differentiation of Th17 cells for anti-tumor responses^46^. Therefore, the physical characteristics of adjuvants, such as size and particle morphology, should also be considered when developing vaccines. Given that many mRNA vaccines are also based on nano-delivery like the LNP systems, it is important to consider the effect of the physical properties of nanoparticles on vaccine efficacy as well as potential undesired inflammatory side effects.

### Adjuvants based on additional immunostimulants

Adjuvants used in mRNA vaccines can also be immunostimulants other than lipid components or mRNA-encoded immune proteins. Arginine-rich protamine peptides can condense and deliver mRNA in vivo by forming a complex with mRNA. The complex formed by protamine and mRNA can further activate TLR7/8 pathways to elicit B-cell and T-cell-dependent responses against influenza A or tumors^47,48^. A protamine-containing delivery platform for mRNA vaccine has been evaluated in clinical trials for treating melanoma, prostate cancer, and non-small-cell lung cancer^49–51^. Another peptide, the cholesterol-modified cationic peptide DP7 (VQWRIRVAVIRK), can activate the TLR2-MyD88-IKK-NF-κB pathway and improve the immune responses evoked by neoantigen vaccine, thereby serving as an effective adjuvant for individualized cancer vaccine^52^. The liposomes with DP7 in mRNA vaccines are efficacious in stimulating DCs maturation, generating CD103^+^ DCs (a major DC subtype for antigen presentation), and secreting proinflammatory cytokines such as TNFα, IL-6, IL-12p70 to facilitate the mRNA vaccine efficacy^53^.

A proof-of-concept adjuvant, palmitic acid-modified TLR7/8 agonist R848 (C16-R848), has been shown to promote the efficiency of mRNA delivery and induce an effective adaptive immune response^16^. A glycolipid antigen, α-GC, which could be presented in the MHC-I-like molecule CD1d on APCs to activate invariant natural killer T (NKT) cells, is also used in mRNA vaccines to boost the antitumor efficacy^54^. Taken together, the immunostimulants demonstrated with adjuvant activities in the mRNA vaccines are functional peptides, ligands for innate immune receptors such as TLRs, and agonists for innate immune cells such as NKT cells. These additional immunostimulants often work by activating the TLRs signaling. Looking for agonists that trigger innate immune pathways independent of TLRs may be worth further exploring as adjuvants in mRNA vaccines.

### The adjuvant components in COVID-19 mRNA vaccines

The pandemic of COVID-2019 has expedited the development of mRNA vaccine research. During the battle against SARS-CoV-2, Moderna and BioNTech vaccines based on pseudouridine-modified mRNA and the LNP delivery system have achieved unprecedented success. The nano-delivery system for mRNA vaccines plays a dual role by acting as a carrier and an adjuvant. In addition, mRNA without pseudouridine modification has the potential self-adjuvant activity to induce inflammatory effects through activating TLRs signaling^23^. However, a recent clinical trial reports that the unmodified mRNA vaccine (CVnCoV) from CureVac showed only 47% efficacy in preventing SARS-CoV-2 infection in phase III trials, which was much weaker than the mRNA-modified vaccines (mRNA-1273 and BNT162b2)^55,56^. Moreover, the study of immunization dose and neutralization levels across CVnCoV, mRNA-1273, and BNT162b2 suggests the lowest neutralization of CVnCoV compared to the other two vaccines when the recommended dose was administered^57^. There are several possible explanations for the different efficacy across three mRNA vaccines. Firstly, the immunogenicity of mRNA plays a critical role in the vaccine efficacy^23,58^. CVnCoV uses an unmodified mRNA construct, which may act as a ligand for PRRs like TLRs to cause inflammatory reactions. A report points out that the mRNA vaccine-induced type I IFN response might establish a temporary state limiting the initial antigen expression, which is harmful to vaccine efficacy^59^. Secondly, the immunization dose of CVnCoV is 12 μg while mRNA-1273 and BNT162b2 are 30 μg and 100 μg, respectively^56,60,61^. The lower mRNA dose of CVnCoV may also weaken the vaccine’s effectiveness.

The mRNA vaccines BNT162b2 and mRNA-1273 are similarly effective in protecting people from COVID-19 infection by different viral variants. Although both vaccines are based on nucleoside-modified mRNA construct and the LNP technology, there are still some differences in how the two vaccines are designed. The administered dosage of mRNA-1273 is lower than BNT162b2. Moreover, mRNA-1273 uses the ionizable lipid SM-102 to achieve a stable nanoparticle structure, while BNT162b2 chooses a different lipid named ALC-0315^62^. Intriguingly, nucleoside-modified mRNA vaccines based on LNP are not immunosilent and have potent immunogenicity^15,28,44^. The immunogenicity of BNT162b2 and mRNA-1273 could generally be attributed to some factors, including the immune recognition of nucleoside-modified mRNA, the adjuvant activity of LNP, and the manufacturing process of vaccine^62^. Modifications of nucleosides can help mRNA escape from the sensing of PRRs like TLRs and RIG-I. Intriguingly, BNT162b2 still activates the MDA-5 signaling pathway to induce type I IFNs to elicit antigen-specific T cell responses and generate neutralizing antibodies^23,28^. LNP could also serve as highly effective adjuvants besides delivery agents^62^. Empty LNP has enhanced B and T cell responses in subunit vaccines against hepatitis B or dengue virus^63,64^. Recently, the ionizable lipid component has been found responsible for LNP-elicited innate immune responses in vivo, as LNP with mRNA can induce the production of chemokines like CXCL10, cytokines IL-1β and IL-6, while empty LNP without the ionizable lipid loses its ability to induce efficient antibody responses^15^. Therefore, the adjuvant activity of LNP may also account for the immunogenicity of mRNA vaccines BNT162b2 and mRNA-1273. In addition, during the preparation of vaccines, dsRNA would be produced as a byproduct during the in vitro transcription (IVT) process when synthesizing mRNA. DsRNA could induce potent inflammatory responses via various intracellular PRRs sensors that contribute to the immunogenicity of mRNA vaccines^65^. These findings suggest that the nucleosides modification of mRNA construct, optimizing the preparation process of antigen-encoding mRNA by purification techniques, and screening for suitable ionizable lipids with delivery and adjuvant activities will be critical for successful mRNA vaccines.

### Summary

In conclusion, the adjuvants in mRNA vaccines can be generally classified into three categories: (1) RNA with self-adjuvant activities from its nucleotide sequences or encoded protein; (2) the components of the delivery system, especially ionizable cationic lipids in LNP; (3) exogenous immunostimulants. Adjuvants can guide the immune response type of mRNA vaccines, thus selecting appropriate adjuvants is essential for designing mRNA vaccines to treat different diseases. An ideal adjuvant for mRNA vaccines should boost the vaccine efficacy by eliciting the desired potent immune response and helping overcome the drawbacks of mRNA, such as low stability and translation efficiency^1^. Ionizable cationic lipids in the LNP system can tremendously impact the efficacy of mRNA vaccines^15,66,67^. It is important to conduct further studies on the cationic lipid components by screening efficient delivery agents with adjuvant activity and low toxicity to prepare mRNA vaccines. Moreover, utilizing mRNA to encode specific functional immune proteins and screening agonists of innate immune receptors as applicable adjuvants are also promising approaches for the adjuvant development of mRNA vaccines. Searching for competent adjuvants will promote a better understanding of mRNA vaccines and help develop safer and more efficacious mRNA vaccines against different diseases.

## Data Availability

The data of this article are available from the corresponding author upon reasonable request.
